# Supplementary material for: Learning curve and surgical outcome of robotic assisted colorectal surgery with ERAS program
Source: Sci Rep. 2022 Nov 29;12:20566. doi: 10.1038/s41598-022-24665-w (PMC9709162; doi:10.1038/s41598-022-24665-w)
Supplement: Supplementary file 1 — Supplementary Information 1. [file 41598_2022_24665_MOESM1_ESM.zip › LSC Raw data-Ñ[▒K/089/H_ñw╝╨░O▒Kñσ.pdf]

正式摘要(電子簽章：000000197573639)

W52 / W52 ☐英文標題(注意:無英文姓名-請補病人基本檔!) ☐【讀取】憑證

臺中榮民總醫院
出院病歷摘要

轉入號 : 轉出號 :

[redacted]

(8)流水編號	
(9)入院日期	109/08/05 (2020) 直腸外科 W52 - 153
(10)轉科日期	
(11)出院日期	109/08/22 (2020) 住院天數計 17 日

診斷

- 入院 :Rectal adenocarcinoma
  - biopsy at Lin-shin H. on 7/08 : adenocarcinoma
  - scope at OPD on 07/28: Colonscopy was up to S-colon(30cm FAV). Rectum tumor was noted at 8cm FAV.
- 出院 :1. Rectal cancer, adenocarcinoma, pT2N2bM0, 8cm FAV
  - s/p Laparoscopic low anterior resection and anastomosis on 2020/08/06,
  - with septic shock with bandemia, suspected anastomosis leakage s/p loop ileostomy on 08/11 s/p Tazocin and Ciprofloxacin (08/11-08/18), Unasyn and Ciprofloxacin (08/19-8/22)

主訴

- Tarry stool for 1 year.

病史

- This 71-year-old male patient denyed any systemic disease came to our hospital due to tarry stool for 1 year. According to patient's statement, he found his stool was sometimes in dark color since 1 year ago without any discomfort. However, he was found stool occult blood positive during health examination at Lin-shin Hospital 1 month ago. He was than received LGI scopy exam with biopsy, which revealed sigmoid colon adenocarcinoma. Therefore, he came to our CRS OPD for help. At OPD, CEA 3.34 ng/ml, CA-199 25.48 U/ml were noted and rectum tumor was found at 8cm FAV by sigmoid colonoscopy on 07/28.
  Under the impression of colorectal cancer, he was admitted to our ward for operation of laparoscopic anterior resection with anastomosis and further management.

體檢發現

- Recto digital examination:FINGER TIP
  Sigmoidoscopy:UP TO 40CM FAV, TUMOR LOCATED 8CM FAV
  Anoscopy:
  Abdomen:
  - General appearance :
    - acute ill looking , conscious clear , without cardiopulmonary distress
    - Vital signs自動帶入date 體溫(BT) 脈搏(P) 呼吸(R) 血壓(BP) 身高(BH) 體重(BW) BMI
      20200805 36.2℃ 70次/分 18次/分 119/77mmHg 169.5cm 65.1kg 22.7
    - Vital signs: B.P. 119/77 mmHg , P.R. 70 /min , R.R. 18 /min , B.T. 36.2 ℃
  - HEENT :
    - Eyes: no anemic conjunctiva, no icteric sclera, normal EOM
    - ENT: no otorrhea, no rhinorrhea, no erythematous mucosa, no injected tonsil, no deformity
  - Neck: supple, freely movable, trachea midline,no deformity, no engorged jugular vein , no palpable thyroid or lymph node enlargement
  - Chest:
    - Inspection: Grossly normal, symmetric and fully expansion, no accessory muscle used, no operation scar, no spider angiomata, no gynecomastia

-
    - Palpation: no palpable mass or nodule, no subcutaneous emphysema, no tenderness
    - Ausculation: clear breathing sound
  - Heart:
    - Palpation: no thrill, no heave
    - Percussion: normal heart size
    - Ausculation: regular heart beat, normal S1, S2, no gallop, no murmur
  - Extremities: No deformity, full ranged of motion without limitation, no clubbing finger or nail cyanosis, no pitting edema, no ulcer or pigmentation, no obvious skin lesion, no varicose vein, no bone, joint or muscle tenderness

CRS CONDITION

- Karnofsky performance status: (90) Able to carry on normal activity; minor signs or symptoms of disease
- PE of Abdomen:
- __1. Inspection: flat
- __2. Ausculation: normoactive bowel sound
- __3. Palapation: soft, no tenderness
- __4. Percussion: tympanic
- CEA: 3.34 ng/ml
- Imaging Studies:
- Colonoscope report: Colonscopy was up to S-colon(30cm FAV). Rectum tumor was noted at 8cm FAV.
- Figures:
  1. colon & rectum

手術日期及方法

- 手術日期及方法20200811 Loop Ileostomy
  20200806 1. Laparoscopic low anterior resection, end-to-end anastomosis, EndoGIA 60/.38mm, CEEA- 29mm. Bilateral doughnut: intact, air tight test: done, no air leak.
  2. High ligation (+) above the left colic artery.
  3. Cut margin: proximal 15 cm, distal 2 cm.
  5. Take down of splenic flexure: (-).
  6. A 12mm penrose drain placed in the pelvis.

住院治療經過

- After admission, laparoscopic LAR was done and the patient tolerate well, liquid diet was tried on 8/7, howevver fever episode was noted on 8/8, where penrose drain was clear, fever servey was done and UTI was suspected. On 8/10, fever episdoe noted again and complicated with septic shock, inatropic was used and empirical abx was used, the penrose drain was still clean, the patient had flattus but mild abd distention. Under the impression of leakage, ileostomy was done on 8/11, after the operation and fluid resuscitation, lab data improved (lactate and band form), diet was tried since 8/13 gradually, LEVOPHED was off since 8/14. The patient tolerate diet well, penrose drain was removed on 8/19. Under stable condition, the patient was discharge with OPD follow up.

合併症

一般檢查

特殊檢查

病理報告

其它

出院時情況

- 3.改門診治療

出院指示

藥名	劑量	劑量單位	用法	頻次	數量	數量單位	現況	天數	單位
Unasyn TAB 375mg	1	TAB	PO	Q12H	14	TAB	TH	7	天
Ultracet tab	1	TAB	PO	Q6HPRN	28	TAB	TH	7	天
12μg Durogesic patch	1	TAP	TOP	Q3D	7	TAP	TH	7	天
(錠)Ciproxin tab 250mg	2	TAB	PO	Q12H	28	TAB	TH	7	天

出院計劃

主治醫師 2062F 蔣鋒帆 / 住院醫師 2332G 林志安

正式摘要**(**電子簽章：**000000197573639)**

W52 / W52

doctorRank:B
